# Effectiveness of a Health Literacy and Diabetes Self-Management Education (DSME) Improvement Program for People With Type 2 Diabetes Mellitus: A Community-Based Quasiexperimental Study in Thailand

**DOI:** 10.1155/jdr/2640702

**Published:** 2025-03-24

**Authors:** Fatima Ibrahim Abdulsalam, Sribud Srichaijaroonpong, Natthida Phoosuwan, Nitikorn Phoosuwan

**Affiliations:** ^1^Department of Community Health, Kasetsart University Chalermphrakiat Sakonnakhon Province Campus, Sakon Nakhon, Thailand; ^2^Department of Public Health and Environment, Nongplapak Sub-district Municipality Office, Nong Khai, Thailand; ^3^Department of Public Health and Caring Sciences, Uppsala University, Uppsala, Sweden

**Keywords:** diabetes mellitus, diabetes self-management education (DSME), health literacy, Northeast Thailand, self-management

## Abstract

**Background:** This community-based randomised controlled study was aimed at evaluating a health literacy (HL) and diabetes self-management education (DSME) improvement program (HL-DSME program) among people with Type 2 diabetes mellitus.

**Methods**: The program conducted in Sakonnakhon Province in northeastern Thailand comprised 1 day of theory and a 4-week period of follow-up. There were 72 participants (36 participants for each intervention and control group) in the program. Independent *t*-tests, one-way analysis of variance and, paired-sample *T*-tests were used to predict HL and self-management scores.

**Results**: The participants in the intervention group increased their total HL score and four dimensions of the HL score significantly in comparison to those in the control group. Total self-management score and the score related to the dimension of exercise behaviour among the participants in the intervention group improved significantly in comparison to those in the control group.

**Conclusion**: The HL-DSME program could increase the total score of HL, scores of four dimensions of HL, the total score of self-management, and the score related to exercise behaviour. Healthcare providers who try to enhance diabetes patients' self-management practices should consider diabetes patients' HL and provide health education regularly. Diabetes management approach should be scalable, efficacious, sustainable, and affordable for patients.

**Trial Registration:** Thai Registry of Clinical Trials: TCTR20241120002

## 1. Background

The International Diabetes Federation (IDF) Atlas reports that in the 21st century, diabetes is one of the fastest-growing global health emergencies [1]. Diabetes has reached an alarming level where today, more than half a billion people worldwide are living with the condition and in the next two decades, this is expected to increase by a rate of 46% [1]. Of all diagnosed diabetes worldwide, Type 2 diabetes mellitus (T2DM) accounts for a vast majority (i.e., more than 90%). In the western Pacific Region, about 1.73 billion people of the adult population are living with diabetes and is adjudged about 38% worldwide [1]. Prevalence of the disease condition is rapidly increasing in the Southeast Asian Region which is predicted to be as high as 152 million T2DM cases by the year 2045 [1]. Currently, in this region, Thailand is one of the top five countries for the number of people living with diabetes, estimated to be about 6.1 million people [1]. If appropriately managed, serious health complications such as cardiovascular diseases (CVDs), nerve damage (neuropathy), kidney damage (nephropathy), lower-limb amputation, and eye disease resulting in visual loss or blindness can be delayed or even prevented. Poor glycemic control or uncontrolled T2DM also leads to acute and chronic complications, and in Thailand, it has been reported recently that predictive factors for uncontrolled T2DM are women, younger age (< 49 years old), residing in the northeastern region, higher BMI, receiving care from community hospitals, amongst others [2]. Some studies conducted in Thailand suggest that majority (~85%) of people living with T2DM had complications [3] and that most people with T2DM need diabetes-related knowledge including general knowledge and insulin-use knowledge [4]. Also, the risk of insufficient knowledge of the disease condition is higher in those with a low level of education or those living in a semiurban area [4].

In different parts of the world, educational intervention studies have proven to be very effective in improving the level of knowledge and self-care practices in DM patients [5–7], contributing tremendous benefits with regard to patients' treatment compliance [8]. Several intervention programs are aimed at preventing complications and helping people with T2DM care for themselves, for example, the National Diabetes Prevention Program (National DPP) [9, 10] and diabetes self-management education (DSME) [11]. The DSME has been used to improve the knowledge, skills, and ability needed for the self-care of diabetes mellitus (DM) patients. This is needed to initiate, maintain, and reinforce essential coping skills and practices required for routine daily care [12]. Although some intervention programs have been implemented in Thailand, such blanket approaches were not as effective in certain regions due to contextual differences like culture, religion, and beliefs of some communities.

To control the progression and prevent long-term health complications, health literacy (HL) and self-care behaviours are important [13] and as such, patients are expected to practice a complex set of these behaviours in their daily routines [11]. In attaining an early detection of the disease and reducing its complications, the role of adequate knowledge of DM and its self-care practices cannot be overemphasized. A patient's knowledge of DM condition may influence positive health outcomes through adequate health education and self-management practices; hence, this study was a community-based study which is aimed at improving HL and self-management among people with T2DM; a health literacy and diabetes self-management education improvement program (HL-DSME program) was developed for this study.

## 2. Methods

This study is aimed at studying the effectiveness of a HL-DSME program among people with T2DM in a community in northeastern Thailand.

### 2.1. Study Setting and Design

This randomised controlled trial study with an allocation ratio of 1:1 was carried out in Sakonnakhon, a northeastern province of Thailand. Sakonnakhon has 18 districts and has a mixture of religions (Christianity, Buddhism, and Islam) and residential areas. Two subdistricts had been selected because they were semiurban areas and were about 15–30 km away from Sakonnakhon city hall. People with T2DM in the Nongsanom-Muang Subdistrict were selected for the intervention group, and those in the Napho-Kusuman Subdistrict were selected for the control group.

### 2.2. Participants

Using a systematic randomisation method, the participants were people with T2DM selected from two subdistricts of Sakonnakhon, that is, Nongsanom-Muang Subdistrict and the Napho-Kusuman Subdistrict. We calculated the sample size [14]. The mean difference score and standard deviations (SDs) for self-management based on our pilot study in a province near Sakonnakhon were 4.0 and 4.3; *β* was 80% and *α* was 0.05. After considering for loss of follow-up, the final number of participants for each group was 36. Inclusion criteria for the intervention group were people diagnosed with T2DM from a physician for more than 1 year, aged 35–69 years, were of Thai nationality, lived in the Nongsanom-Muang Subdistrict for more than 1 year, and agreed to participate in the study. Exclusion criteria were having reported mental health and refusing to participate in the entire study.

Inclusion criteria for the control group were people diagnosed with T2DM from a physician for more than 1 year, aged 35–69 years, were of Thai nationality, lived in Napho-Kusuman Subdistrict for more than 1 year, and agreed to participate in the study. The age range (35–69 years) was selected because T2DM is most prevalent among middle-aged and older adults, and this age group is more likely to benefit from DSME programs [1]. Exclusion criteria were having reported mental health and refusing to participate in the entire study.

**Figure 1:**
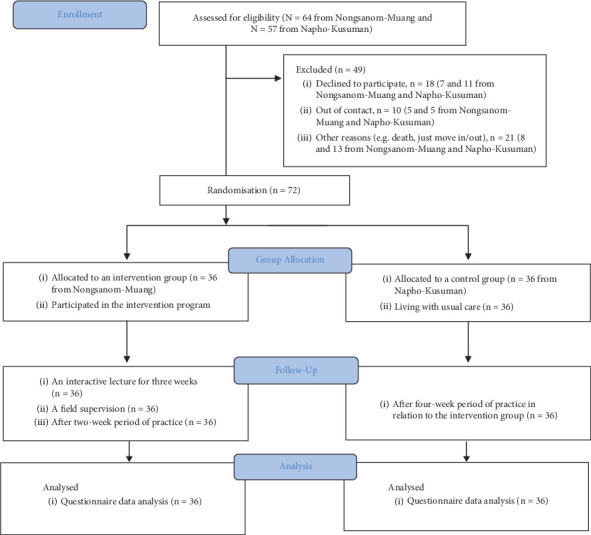
Flow diagram of the study.

A total of 64 individuals with T2DM from the Nongsanom-Muang Subdistrict and 57 individuals from the Napho-Kusuman Subdistrict were initially eligible for the study. However, some were excluded due to various reasons, such as passing away or a lack of interest in participating. Using a systematic randomisation method, the researchers enrolled and assigned the participants to the intervention and control groups. The assigned participants agreed to participate in the groups they were assigned, and the study was carried out from March to April 2023 (see Figure 1).

### 2.3. HL-DSME Program

The HL-DSME program was developed by the study researchers based on the theory of HL [15] and self-management [16]. It contained three parts: (1) an interactive lecture (three sessions during 3 weeks, 60 min for each session), (2) a 2-week period of practice, and (3) one field supervision (see Table 1).

#### 2.3.1. An Interactive Lecture

This part had three sessions for 3 weeks on Saturdays each week based on the convenience of the participants. Each session lasted for 60 min.

The first session covered contents related to skills to access and cognitive skills about T2DM. The innovative booklet was used to make the participants gain knowledge of T2DM and diabetes complications (this activity is called “Do not entrust on the diabetes”). The participants were taught and demonstrated how to search for valid information from online applications (this activity is called “Searching on valid information”). Thereafter, they practised searching on the Internet under the supervision of the researcher.

The second session provided three contents related to (1) reminding participants, (2) communication skills, and (3) media literacy. Reminding participants was done by the researcher to remind them what they had learnt and what they had practised during the week. Communication skills are based on how the participants can communicate their diabetes with healthcare professionals and their relatives, including how to read their prescriptions and drug administration (this activity is called “Improve understanding about diabetes”). This communication skill used an individual booklet provided by the researcher. Media literacy was improved by using how to use media and useful information and sources of health information and how to check health data from online sources (this activity is called “Perception on creative media”).

The third session was about self-management skills and decision skills. The initial meeting focused on reminding the participants how to check valid information online and their practices during the previous week. The self-management skill involved a knowledge whiteboard; the individual book provided from the latter week; and teaching how people with T2DM can manage themselves with food, physical exercises, and exchange ideas for food consumption and exercises at the final minutes (this activity is called “Away from diabetes”). Decision skill was an activity to improve people with T2DM for their decision-making, how to access health information, and health perception communication. It also covered a workshop where the participants made decisions for good self-management by putting a star symbol on good self-management practice, for example, doing exercises for more than 150 min each week (this activity is called “Think for management of diabetes”). A demonstration of a good exercise for people with T2DM was provided in this session, where the participants could choose an exercise that was appropriate to their ability (this activity is called “Exercise and try to live with diabetes”). Finally, the participants had a chance to practice themselves in class.

#### 2.3.2. A 2-Week Period of Practice

After the third session, the participants were asked to practice by themselves self-management food consumption and physical exercises. They were requested to note their practices in their booklets.

#### 2.3.3. Field Supervision

It was conducted once during the 2-week period of practice by the researcher. The researcher and each participant agreed on a date, place, and time the researcher could go to provide supervision on their practices and to monitor the participant's practices. If requested from a participant for additional supervision, one more time was supported by telephone or online supervision.

### 2.4. Instruments

A questionnaire consisting of three parts: (1) sociodemographics and disease history of participants, (2) HL related to people with T2DM, and (3) self-management related to people with T2DM, was developed by the researchers. The questionnaire was validated to examine what it is intended to do and appropriately framed to include all necessary aspects. Content validity was confirmed by three PhD experts via the index of item objective congruence (IOC) = 0.5–1.0 for each item. A reliability test was done among 30 people with T2DM who did not participate in the study, and the overall Cronbach alpha coefficient was 0.723.

The sociodemographic part gathered information about sex, age, religion, marital status, education level, occupation, income/month, duration of living with diabetes, family history of diabetes, alcohol consumption behaviour, smoking behaviour, physical exercise behaviour, weight, height, and waist circumference. Standard protocols and techniques for data collection and measurements were observed according to recommended population-specific values [17, 18]. The HL part was created by the Health Education Division, Ministry of Public Health, Thailand, in 2015 [19]. It had six dimensions related to diabetes: (1) knowledge and understanding of the disease, (2) accessibility to healthcare services, (3) health communication and risk management, (4) management of disease conditions, (5) disease perception and e-Health, and (6) decision-making for self-management. The part on knowledge and understanding of the disease had seven multiple-choice questions; each question provided a score of 1 if answered correctly or 0 if answered incorrectly. Therefore, the score of this part ranged between 0 and 7. The parts of accessibility to healthcare services, health communication and risk management, management of disease conditions, disease perception, and e-Health comprised four questions for each part, where each question had four options to evaluate the frequency of access to healthcare services (i.e., 3 = *always*, 2 = *often*, 1 = *sometimes*, and 0 = *never*). Therefore, each part could score between 0 and 12. The part of decision-making for self-management had two multiple-choice questions; each question answered correctly scores 1, and if answered incorrectly scores 0. The score of this part then ranged between 0 and 2. The self-management part was assessed in three dimensions related to the frequency of food consumption behaviour (four questions), exercise behaviour (three questions), and medication behaviour (three questions) weekly according to the participants. Therefore, each question ranged between 0 and 7; the scores for food consumption behaviour, exercise behaviour, and medication behaviour, ranged between 0 and 28, 0 and 21, and 0 and 21, respectively.

### 2.5. Ethical Consideration

The Ethics Committee at Kasetsart University approved the proposal (COA65/004) and the study was registered as a clinical trial. Directors of the two selected subdistrict health promotion hospitals approved the HL-DSME program and agreed to data collection. Oral explanation of the research was given to all participants who signed consent forms before participation. Participants' privacy and confidentiality were duly ensured as data was codified to maintain anonymity. All the information obtained was guaranteed anonymity.

### 2.6. Data Analysis

A statistical software package was used to analyse data, and study variables and participants' demographics were presented as percentages, means, and SDs. Independent *t*-tests and one-way analysis of variance (ANOVA) were used to assess differences and associations among demographic factors, lifestyle habits, HL scores, and self-care practice scores. Additionally, a linear regression model was applied to evaluate whether the intervention program influenced HL and self-management scores. The effect of the intervention was measured using beta coefficients and 95% confidence intervals (CIs).

In the first regression model, demographic variables (age, gender, marital status, education, religion, occupation, and average monthly income), disease history (years since diagnosis and family history of DM), and lifestyle factors (exercise, waist circumference, body mass index, smoking, and alcohol consumption) were included as independent variables to predict HL scores. The same set of variables was used in the second regression model to predict self-management scores. All categorical variables, including age, gender, marital status, education level, occupation, years of disease diagnosis, family history of diabetes, exercise habits, waist circumference, body mass index, income level, and addictive behaviours (smoking and alcohol consumption), were dummy coded before analysis. Each dimension of the outcome variables, that is, HL and self-management were also analysed. For nonviolation of assumptions, normality and independent assumptions of the regression analysis were examined through standardized residual plots and collinearity statistics.

## 3. Results

The mean age was 55.76 (±6.13) years with the majority (56.9%) within the age range of 50–59 years. Almost all of our participants (86.1%) had a primary school level of education and are Buddhists (70.8%). About 40.3% have been diagnosed with DM for a decade or more. Few have a history of a family member diagnosed with DM (27.8%) and exercise regularly (40.3%), but many have a larger waist circumference greater than the World Health Organization (WHO) cut-off standards (70.8%) (see Table 2).

As for the HL and self-management scores and sociodemographic characteristics of the study participants of both intervention and control groups, they seemed to be similar except for some variables like religion, years of DM diagnosis, waist circumference, the HL mean posttest score, and self-management scores of both groups (see Table 3).

The adjusted analysis revealed a significant increase in HL scores among participants in the intervention group (*β* = 0.46, 95% CI: 0.37–0.72, *p* = 0.001). However, the intervention program did not significantly impact self-management scores, as there was no notable difference between the intervention and control groups (*β* = 0.21, 95% CI: −0.37 to 2.56, *p* = 0.14) (see Table 4).

For each dimension in the HL score, results showed that participants in the intervention group increased their HL score for Dimensions 1, 2, 4, and 5 compared to the control group (*β* = 0.32, 95% CI 0.24–1.08, *p* = 0.006; *β* = 0.47, 95% CI 0.32–0.68, *p* < 0.001; *β* = 0.3, 95% CI 0.29–0.63, *p* = 0.038; and *β* = 0.33, 95% CI 0.09–0.50, *p* = 0.023, respectively) (see Table 5).

Compared to the control group, participants in the intervention group had statistically significant higher self-management scores only in Dimension 2 (*β* = 0.31, 95% CI 0.16–0.52, *p* = 0.027) (see Table 6).

A paired-sample *t*-test was performed to compare the mean scores of DM participants in the intervention and control groups before and after the intervention. A significant improvement in HL scores was observed between pre- and postintervention assessments in both groups. Specifically, the intervention group showed a significant increase (mean = 50.53, SD = 5.09 vs. mean = 37.22, SD = 5.53; *t*(35) = 13.31, *p* < 0.001), while the control group also demonstrated a smaller but significant improvement (mean = 43.14, SD = 5.69 vs. mean = 35, SD = 6.67; *t*(35) = 8.14, *p* < 0.001). Regarding self-management scores, a significant increase was observed only in the intervention group (mean = 23.56, SD = 2.22 vs. mean = 17.67, SD = 3.83; *t*(35) = 5.89, *p* < 0.001), whereas no significant change was detected in the control group (see Table 7).

## 4. Discussion

This study found that the HL-DSME program significantly improved HL scores and self-management behaviours among individuals with T2DM. Specifically, participants in the intervention group demonstrated a significant increase in overall HL scores, with notable improvements in four of the six HL dimensions. Additionally, self-management scores related to exercise behaviour improved significantly in the intervention group compared to the control group. However, no significant differences were observed in self-management behaviours related to food consumption and medication adherence between the two groups. These findings suggest that targeted educational interventions can effectively enhance HL and specific aspects of self-management, particularly exercise, among individuals with T2DM.

In both groups, there was a female preponderance similar to other findings [12, 20, 21]. The mean outcome variable scores of both groups were significantly different as HL and self-management scores were positively changed by the intervention program. Reports suggest that there is a significant pathway from HL to self-care activities [22, 23]; that is, the willingness of patients to carry out health actions in managing their health is related to the receptiveness of HL. Diabetes knowledge such as its risks and illness perception is influenced by HL which can result in decisions about whether or not a patient attempts to perform diabetes self-care management practices [24]. In our study, participants in the intervention group who were of age 60 and above seemed more receptive to the knowledge obtained during the intervention program; perhaps, it strengthened their belief in their capabilities to organize and carry out these self-care practices required to manage the disease condition. Further qualitative research study should be conducted to explore beliefs and perceptions related to diabetes HL and self-care management in the community.

HL is inherently related to increased glycemic control and diabetes knowledge, although it is not investigated for some dimensions, for example, communicative HL [25]. However, this detailed study conducted a deeper investigation and found that overall HL improved in four specific dimensions: knowledge and understanding of the disease (Dimension 1), accessibility to healthcare services (Dimension 2), management of disease conditions (Dimension 4), and disease perception and e-Health (Dimension 5) within 5 weeks of the intervention. However, no significant improvements were observed in health communication and risk management (Dimension 3) or decision-making for self-care management (Dimension 6). The intervention may have highly influenced subjectiveness to self-care management decisions to adopt healthy behaviours. It may have expanded their compliance and management towards the disease condition [24]. An extension of a follow-up period for the HL-DSME program [26] and including more individual programs could improve the HL score for the two dimensions mentioned above [27].

Self-care practices related to Dimension 2 (exercise behaviour) significantly improved in the intervention group. The participants in the intervention group were more likely to actively engage in self-management and achieve optimal health outcomes. Nowadays, one of the first management strategies recommended for newly diagnosed T2DM patients is regular exercise together with diet and behaviour modification. Be it aerobic, resistance training, or a combination, exercise training facilitates improved glucose regulation which is a central component for preventing all T2DM [28] and as such, healthcare providers need to make this diabetes management approach scalable, efficacious, sustainable, and affordable for their patients. In addition, our intervention program provided a lecture in the third week to improve participants' understanding of how they can select a suitable exercise according to their capability. Thereafter, they had 2 weeks to practice by themselves with supervision from the researcher. This might increase their exercise behaviour and confidence to exercise in the long run.

However, self-management for food consumption and medication behaviours showed no differences between participants in the intervention and control groups similar to a study [29], but in contrast with another [30]. In general, behaviours related to food consumption and medication may change after 2 or 3 months as people with T2DM may adopt new behaviours due to routine check-ups with their physician [27, 31]. For behavioural and clinical outcomes, the HL-DSME program could be more beneficial if conducted for more than 3 months.

The strength of the study partly lies in its design in which, as a randomised controlled trial, biases are managed [32]. With the application of a sample size calculation and sample randomisation, our findings could be representable and generalized to other people with T2DM having similar characteristics in other Thailand provinces. For the study, individual booklets following the HL-DMSE guidelines were provided to all participants in the intervention group and the intervention improved HL through self-education. These booklets (focusing on person-centred care) were designed respectively and responsively to patient preferences, needs, and values to ensure that all clinical decisions are guided by patient values; however, the study has its limitations. It was conducted within a short period of time with a short follow-up; perhaps, a longer period might yield better results; also, it was conducted in the northeastern region of Thailand which has a low socioeconomic index [33] as other regions (with much higher socioeconomic index) may differ.

## 5. Conclusions

To control the progression and prevent long-term health complications, HL and self-care behaviours are important for people diagnosed with T2DM as they are expected to practice a complex set of daily routines related to the disease condition. Due to the HL-DSME program, HL in several dimensions and self-management related to exercise behaviour significantly improved in the intervention group. Healthcare providers who try to enhance diabetes patients' self-management practices should consider diabetes patients' HL and provide health education regularly. Diabetes management approach should be scalable, efficacious, sustainable, and affordable for patients.

## Figures and Tables

**Table 1:** Contents of the health literacy and diabetes self-management education (HL-DSME) program in relation to the six skills of health literacy.

**Skill**	**Contents**	**Implementation**
Access	The “Do not entrust on diabetes” activity made participants gain knowledge related to T2DM and diabetes complications	First session of an interactive lecture by a registered public health official, with the support of an innovative booklet
		Seven-day period after the first session

Cognitive	The “Searching on valid information” activity shared and taught participants how to search for valid information from an online application	First session of interactive led by a registered public health official, practised in groups under supervision from the official
		Practice within 7 days after the first session

Communication	The “Improve understanding about diabetes” activity was based on how the participants can communicate their condition with healthcare professionals and their relatives, including how to read their prescription and drug administration. An individual booklet provided by the researcher was used	Second session included the use of their booklets, and participants were taught by a registered public health official
		Reminding participants on how to check valid information online, at the beginning of the third session.

Media	The “Perception on creative media” activity was aimed at improving how to use media and useful information and source of health information and how to check health data from online sources	Demonstration and practice in groups under the supervision of the registered public health official
		Discussing with participants about their practices during the previous week at the beginning of the third session

Self-management	“Away from diabetes” activity was aimed at teaching people with T2DM to manage themselves with food and physical exercises	Third session was conducted by the registered public health official using a knowledge whiteboard, and the individual booklet was used. Exchange idea activity for food consumption and exercises at the final minutes was done
		Practice for 2 weeks after the third session for self-management food consumption, and physical exercises were advised. A note of the practices in their booklet was requested

Decision	The “Think for management of diabetes” activity was aimed at improving people with T2DM for their decision-making, how to access health information, and health perception communication The “Exercise and try to live with diabetes” activity was aimed at making participants practice themselves in a class	A workshop for good self-management by putting a star symbol on good self-management was covered
		A demonstration of a good exercise for people with T2DM was provided, where the participants could choose an exercise that was appropriate to their own ability
		Supervision of the participants through a face-to-face visit

Abbreviation: T2DM, Type 2 diabetes mellitus.

**Table 2:** Characteristics of study participants.

**Category**	**Subcategory**	**n** ** (per cent)**
Age (years)	< 50	13 (18.1)
	50–59	41 (56.9)
	60 and above	18 (25)

Mean = 55.76 ± 6.13, range = 38–68		

Gender	Male	13 (18.1)
	Female	59 (81.9)

Marital status	Married	60 (83.3)
	Not married	12 (16.7)

Education	Primary school	62 (86.1)
	Secondary school or more	10 (13.9)

Religion	Buddhism	51 (70.8)
	Christianity	21 (29.2)

Occupation	Farmer	60 (83.3)
	Nonfarmer	12 (16.7)

Years of DM diagnosis (years)	Less than 5	21 (29.2)
	5–9	22 (30.6)
	≥ 10	29 (40.3)

Family history of DM	No	52 (72.2)
	Yes	20 (27.8)

Regular exercise	No	43 (59.7)
	Yes	29 (40.3)

Waist circumference (cm)	Standard	21 (29.2)
	Greater than standard	51 (70.8)

BMI (kg/m^2^)	Normal weight	34 (47.2)
	Overweight	38 (52.8)

Mean = 25.08 ± 4.14, range = 16.89–39.00		

Monthly income (US$)	≤ 30	21 (29.2)
	3–150	42 (58.3)
	≥ 150	9 (12.5)

Smoking	Never	63 (87.5)
	Quit smoking	5 (6.9)
	Currently smoking	4 (5.6)

Alcohol consumption	Never	51 (70.8)
	Quit alcohol	8 (11.1)
	Currently take alcohol	13 (13)

Abbreviations: BMI, body mass index; DM, diabetes mellitus.

**Table 3:** Sociodemographic characteristics of participants.

**Characteristics**	**Intervention group (** **n** = 36**)**	**Control group (** **n** = 36**)**	**p** ** value**
Age (years)			
Mean (SD)	55.47 (5.05)	56.06 (7.11)	0.689^a^
Gender, *n* (%)			
Male	6 (16.7)	7 (19.4)	0.759^b^
Female	30 (83.3)	29 (80.6)	
Marital status, *n* (%)			
Married	32 (88.9)	28 (77.8)	0.206^b^
Not married	4 (11.1)	8 (22.2)	
Education level, *n* (%)			
Primary school	29 (80.6)	33 (91.7)	0.173^b^
Secondary school or more	7 (19.4)	3 (8.3)	
Religion, *n* (%)			
Buddhism	16 (44.4)	35 (97.2)	**< 0.001** ^b^
Christianity	20 (55.6)	1 (2.8)	
Occupation, *n* (%)			
Farmer	31 (86.1)	29 (80.6)	0.527^b^
Nonfarmer	5 (13.9)	7 (19.4)	
Years of DM diagnosis (years), *n* (%)			
Less than 5	12 (33.3)	9 (25)	**0.036** ^b^
5–9	6 (16.7)	16 (44.4)	
10 and above	18 (50)	11 (30.6)	
Family history of DM, *n* (%)			
No	22 (61.1)	30 (83.3)	0.064^c^
Yes	14 (38.9)	6 (16.7)	
Regular exercise, *n* (%)			
No	25 (69.4)	18 (50)	0.149^c^
Yes	11 (30.6)	18 (50)	
Waist circumference (cm), *n* (%)			
Standard	6 (16.7)	15 (41.7)	**0.020** ^b^
Greater than standard	30 (83.3)	21 (58.3)	
BMI (kg/m^2^), *n* (%)			
Normal weight	15 (41.7)	19 (52.8)	0.345^b^
Overweight	21 (58.3)	17 (47.2)	
Monthly income (US$), *n* (%)			
≤ 30	10 (27.8)	11 (30.6)	0.924^b^
31–150	21 (58.3)	21 (58.3)	
≥ 150	5 (13.9)	4 (11.1)	
Smoking, *n* (%)			
Never	33 (91.7)	30 (83.3)	0.511^b^
Quit smoking	2 (5.6)	3 (8.3)	
Currently smoking	1 (2.8)	3 (8.3)	
Alcohol, *n* (%)			
Never	23 (63.9)	28 (77.8)	0.277^b^
Quit alcohol	6 (16.7)	2 (5.6)	
Currently take alcohol	7 (19.4)	6 (16.7)	
Health literacy			
Pretest score mean (SD)	37.22 (5.53)	35.00 (6.67)	0.128^a^
Posttest score mean (SD)	50.53 (5.09)	43.14 (5.69)	**< 0.001** ^a^
Self-management			
Pretest score mean (SD)	17.67 (3.83)	20.92 (3.53)	**< 0.001** ^a^
Posttest score mean (SD)	23.56 (2.22)	21.81 (2.64)	**0.003** ^a^

*Note:* Values in bold indicate statistical significance at 0.05 level.

Abbreviations: BMI, body mass index; DM, diabetes mellitus; SD, standard deviation.

^a^Obtained by *T*-test.

^b^Obtained by chi-square test.

^c^Obtained by Fisher's exact test.

⁣^∗^Statistical significance at 0.05 level.

**Table 4:** Linear regression model for comparison of posttest scores between intervention and control groups (*n* = 72).

**Category**	**Health literacy score**		**Self-management score**	
	**Crude analysis coefficient ** **B** ** (95% CI)**	**Adjusted analysis coefficient ** **B** ** (95% CI)**	**Crude analysis coefficient ** **B** ** (95% CI)**	**Adjusted analysis coefficient ** **B** ** (95% CI)**
Age	0.38 (0.14, 0.57)⁣^∗^	0.17 (−0.47, 5.03)	0.24 (0.04, 2.52)⁣^∗^	0.13 (−0.57, 1.91)
Gender	0.07 (−2.80, 5.21)	—	0.14 (−0.65, 2.50)	—
Marital status	−0.18 (−7.18, 0.98)	—	−0.13 (−2.54, 0.71)	—
Education	0.13 (−2.03, 6.83)	—	0.11 (−0.92, 2.59)	—
Religion	0.36 (0.13, 0.55)⁣^∗^	0.046 (−2.82, 4.12)	0.30 (0.14, 0.44)⁣^∗^	0.14 (−0.77, 2.33)
Occupation	−0.11 (−6.02, 2.22)	—	−0.19 (−2.92, 0.29)	—
Years of DM diagnosis	−0.21 (−6.91, 0.94)	—	−0.12 (−2.24, 0.90)	—
Family history of DM	0.23 (0.001, 0.43)⁣^∗^	0.09 (−1.68, 4.25)	0.09 (−0.85, 1.87)	—
Regular exercise	0.04 (−2.64, 3.66)	—	0.22 (−0.04, 2.38)	—
Waist circumference	0.21 (−0.33, 6.32)	—	0.10 (−0.78, 1.89)	—
BMI	0.04 (−2.52, 3.67)	—	0.01 (−1.16, 1.29)	—
Monthly income	−0.09 (−4.67, 2.29)	—	0.23 (−0.19, 2.53)	—
Smoking	−0.09 (−8.43, 3.74)	—	−0.17 (−4.04, 0.72)	—
Alcohol consumption	0.07 (−3.64, 6.35)	—	0.39 (−1.67, 2.29)	—
Intervention	0.57 (0.38, 0.72)⁣^∗^	0.46 (0.37, 0.72)⁣^∗^	0.34 (0.10, 0.56)⁣^∗^	0.21 (−0.37, 2.56)

*Note:* Dependent variable is posttest scores.

Abbreviations: BMI, body mass index; CI, confidence interval; DM, diabetes mellitus.

⁣^∗^Statistically significant at 0.05 level.

**Table 5:** Regression analysis for posttest scores in health literacy dimensions.

**Category**	**Health literacy score**											
	**Dimension 1**		**Dimension 2**		**Dimension 3**		**Dimension 4**		**Dimension 5**		**Dimension 6**	
	**Crude analysis coefficient ** **B** ** (95% CI)**	**Adjusted analysis coefficient ** **B** ** (95% CI)**	**Crude analysis coefficient ** **B** ** (95% CI)**	**Adjusted analysis coefficient ** **B** ** (95% CI)**	**Crude analysis coefficient ** **B** ** (95% CI)**	**Adjusted analysis coefficient ** **B** ** (95% CI)**	**Crude analysis coefficient ** **B** ** (95% CI)**	**Adjusted analysis coefficient ** **B** ** (95% CI)**	**Crude analysis coefficient ** **B** ** (95% CI)**	**Adjusted analysis coefficient ** **B** ** (95% CI)**	**Crude analysis coefficient ** **B** ** (95% CI)**	**Adjusted analysis coefficient ** **B** ** (95% CI)**
Age	0.29 (−0.03, 1.20)	—	0.25 (−0.28, 2.50)	—	0.27 (−0.13, 1.90)	—	−0.20 (−1.74, 0.16)	—	0.35 (0.21, 3.67)⁣^∗^	0.33 (0.19, 3.55)⁣^∗^	−0.10 (−0.47, 0.20)	—
Gender	−0.002 (−0.63, 0.62)	—	0.13 (−0.62, 2.09)	—	0.03 (−0.91, 1.12)	—	−0.03 (−1.83, 1.45)	—	0.12 (−0.73, 2.26)	—	−0.08 (−0.78, 0.37)	—
Marital status	−0.23 (−1.24, −0.009)⁣^∗^	−0.18 (−1.09, 0.12)	−0.10 (−1.99, 0.82)	—	−0.08 (−1.38, 0.71)	—	−0.15 (−2.73, 0.63)	—	−0.06 (−1.97, 1.13)	—	−0.04 (−0.69, 0.49)	—
Education	0.13 (−0.30, 1.08)	—	0.13 (−0.68, 2.33)	—	0.15 (−0.39, 1.84)	—	0.22 (−0.11, 3.46)	—	−0.17 (−2.83, 0.47)	—	−0.01 (−0.68, 0.60)	—
Religion	0.21 (−0.05, 0.98)	—	0.31 (0.51, 2.50)⁣^∗^	0.02 (−1.13–1.34)	0.05 (−0.68, 1.04)	—	0.34 (0.14, 0.51)⁣^∗^	0.13 (−0.77, 2.25)	0.22 (−0.05, 2.43)	—	−0.12 (−0.72, 0.25)	—
Occupation	−0.08 (−0.86, 0.43)	—	−0.10 (−1.99, 0.82)	—	−0.18 (−1.76, 0.30)	—	−0.02 (−1.85, 1.55)	—	−0.10 (−2.16, 0.93)	—	0.16 (−0.19, 0.99)	—
Years of DM diagnosis	−0.17 (−0.93, 0.24)	—	−0.12 (−1.94, 0.76)	—	−0.24 (−0.47, −0.01)⁣^∗^	—	−0.29 (−0.49, −0.04)⁣^∗^	−0.15 (−2.09, 0.42)	0.35 (0.01, 0.40)⁣^∗^	0.12 (−0.56, 1.72)	−0.14 (−0.85, 0.30)	—
Family history of DM	0.18 (−0.13, 0.92)	—	0.22 (−0.09, 2.20)	—	0.20 (−0.13, 1.58)	—	0.26 (0.07, 0.41)⁣^∗^	0.14 (−0.48, 2.13)	−0.08 (−1.72, 0.85)	—	0.05 (−0.40, 0.59)	—
Regular exercise	0.02 (−0.44, 0.54)	—	−0.01 (−1.11, 1.04)	—	0.08 (−0.54, 1.05)	—	−0.09 (−1.76, 0.81)	—	0.17 (−0.32, 2.01)	—	−0.07 (−0.58, 0.33)	—
Waist circumference	0.09 (−0.32, 0.73)	—	0.23 (0.06, 2.25)⁣^∗^	0.10 (−0.56, 1.52)	0.04 (−0.70, 1.01)	—	0.25 (0.01, 0.47)⁣^∗^	0.11 (−0.64, 1.90)	0.08 (−0.85, 1.69)	—	−0.18 (−0.85, 0.11)	—
BMI	0.17 (−0.14, 0.81)	—	−0.04 (−1.24, 0.86)	—	−0.01 (−0.81, 0.75)	—	0.08 (−0.84, 1.68)	—	−0.05 (−1.41, 0.91)	—	0.16 (−0.14, 0.74)	—
Monthly income	0.14 (−0.61, 1.02)	—	−0.06 (−1.46, 0.93)	—	−0.14 (−1.35, 0.40)	—	0.11 (−0.56, 1.45)	—	−0.08 (−2.36, 1.57)	—	−0.02 (−0.39, 0.32)	—
Smoking	−0.10 (−1.36, 0.54)	—	−0.13 (−3.17, 0.95)	—	−0.07 (−1.98, 1.10)	—	−0.20 (−5.07, 0.39)	—	−0.13 (−3.50, 1.03)	—	0.23 (0.04, 0.42)⁣^∗^	—
Alcohol	0.09 (−0.50, 1.05)	—	0.05 (−1.33, 2.07)	—	0.03 (−1.14, 1.40)	—	0.01 (−0.78, 0.84)	—	0.10 (−0,91, 2.15)	—	0.17 (−0.08, 0.48)	—
Intervention	0.35 (0.27, 1.14)⁣^∗^	0.32 (0.24, 1.08)⁣^∗^	0.51 (1.31, 3.09)⁣^∗^	0.47 (0.32, 0.68)⁣^∗^	0.09 (−0.50, 1.05)	—	0.48 (0.32, 0.62)⁣^∗^	0.30 (0.29, 0.63)⁣^∗^	0.30 (0.07, 0.51)⁣^∗^	0.33 (0.09, 0.50)⁣^∗^	0.09 (−0.27, 0.61)	—

*Note:* Dependent variable is posttest scores in every dimension; Dimension 1, knowledge and understanding of the disease; Dimension 2, accessibility to healthcare services; Dimension 3, health communication and risk management; Dimension 4, management of disease conditions; Dimension 5, disease perception and e-Health; Dimension 6, decision-making for self-management.

Abbreviations: BMI, body mass index; CI, confidence interval; DM, diabetes mellitus.

⁣^∗^Statistically significant at 0.05 level.

**Table 6:** Regression analysis for posttest scores in self-management dimensions.

**Self-management score**						
**Category**	**Dimension 1**		**Dimension 2**		**Dimension 3**	
	**Crude analysis coefficient ** **B** ** (95% CI)**	**Adjusted analysis coefficient ** **B** ** (95% CI)**	**Crude analysis coefficient ** **B** ** (95% CI)**	**Adjusted analysis coefficient ** **B** ** (95% CI)**	**Crude analysis coefficient ** **B** ** (95% CI)**	**Adjusted analysis coefficient ** **B** ** (95% CI)**
Age	0.09 (−0.19, 0.44)	—	0.32 (0.10, 0.54)⁣^∗^	0.18 (−0.19, 1.69)	−0.24 (−0.46, 0.07)	—
Gender	0.15 (−0.20, 0.87)	—	0.06 (−0.97, 1.63)	—	0.25 (0.02, 0.51)⁣^∗^	—
Marital status	0.02 (−0.51, 0.61)	—	−0.19 (−2.39, 0.25)	—	0.09 (−0.16, 0.36)	—
Education	0.12 (−0.30, 0.90)	—	0.08 (−0.99, 1.90)	—	0.07 (−0.20, 0.36)	—
Religion	0.07 (−0.31, 0.60)	—	0.29 (0.13, 0.44)⁣^∗^	0.05 (−0.93, 1.42)	0.19 (−0.04, 0.38)	—
Occupation	−0.02 (−0.61, 0.51)	—	−0.24 (−2.67, −0.06)⁣^∗^	−0.16 (−2.10, 0.25)	0.09 (−0.16, 0.36)	—
Years of DM diagnosis	0.01 (−0.24, 0.27)	—	0.13 (−0.28, 0.92)	—	−0.20 (−0.43, 0.07)	—
Family history of DM	−0.17 (−0.80, 0.12)	—	0.18 (−0.28, 1.92)	—	0.03 (−0.19, 0.24)	—
Regular exercise	−0.04 (−0.49, 0.35)	—	0.29 (0.09, 0.48)⁣^∗^	0.30 (0.07, 0.49)⁣^∗^	−0.01 (−0.21, 0.19)	—
Waist circumference	−0.04 (−0.53, 0.38)	—	0.13 (−0.49, 1.69)	—	0.04 (−0.18, 0.25)	—
BMI	−0.08 (−0.56, 0.27)	—	0.05 (−0.81, 1.19)	—	0.02 (−0.18, 0.21)	—
Monthly income	0.14 (−0.13, 0.53)	—	0.26 (0.04, 0.43)⁣^∗^	0.09 (−0.53, 1.28)	0.15 (-0.14, 0.52)	—
Smoking	−0.20 (−1.51, 0.11)	—	−0.11 (−2.89, 1.05)	—	−0.05 (−0.52, 0.34)	—
Alcohol	−0.23 (−1.06, 0.02)	—	0.08 (−1.06, 2.18)	—	−0.18 (−0.55, 0.07)	—
Intervention	0.08 (−0.28, 0.55)	—	0.36 (0.15, 0.55)⁣^∗^	0.31 (0.16, 0.52)⁣^∗^	0.14 (−0.08, 0.30)	—

*Note:* Dependent variable is posttest scores in every dimension. Dimension 1, frequency of food consumption behaviour; Dimension 2, exercise behaviour; Dimension 3, medication behaviour.

Abbreviations: BMI, body mass index; CI, confidence interval; DM, diabetes mellitus.

⁣^∗^Statistically significant at 0.05 level.

**Table 7:** Difference in health literacy and self-management pretest and posttest scores of DM participants in the group with or without intervention.

**Outcome variable**	**Group**	**Mean score (±SD)**		**Paired-sample mean (95% CI)**	**p** ** value**
		**Posttest score**	**Pretest score**		
Health literacy	Intervention	50.53 (5.09)	37.22 (5.53)	13.31 (11.09, 15.52)	< 0.001⁣^∗^
	Control	43.14 (5.69)	35.00 (6.67)	8.14 (5.31, 10.97)	< 0.001⁣^∗^
Self-management	Intervention	23.56 (2.22)	17.67 (3.83)	5.89 (4.53, 7.24)	< 0.001⁣^∗^
	Control	21.81 (2.64)	20.92 (3.52)	0.89 (−0.36, 2.14)	0.157

*Note:* Asterisk (⁣^∗^) means statistically significant at 0.05 level.

Abbreviations: CI = confidence interval, DM = diabetes mellitus; SD = standard deviation.

## Data Availability

The data are available on request from the authors. The data that support the findings of this study are available from the corresponding author upon reasonable request.
